# Cannabidiol affects breast meat volatile compounds in chickens subjected to different infection models

**DOI:** 10.1038/s41598-022-23591-1

**Published:** 2022-11-07

**Authors:** Paweł Konieczka, Iwona Wojtasik-Kalinowska, Andrzej Poltorak, Misza Kinsner, Dominika Szkopek, Bartosz Fotschki, Jerzy Juśkiewicz, Joanna Banach, Monika Michalczuk

**Affiliations:** 1 Department of Animal Nutrition, The Kielanowski Institute of Animal Physiology and Nutrition, Polish Academy of Sciences, Instytucka 3, 05-110 Jabłonna, Poland; 2 Department of Poultry Science and Apiculture, University of Warmia and Mazury, Oczapowskiego 5, 10-718 Olsztyn, Poland; 3 Department of Technique and Food Development, Warsaw University of Life Sciences, 159C Nowoursynowska, 02-776 Warsaw, Poland; 4 Institute of Animal Reproduction and Food Research, Polish Academy of Sciences, Tuwima 10, 10-748 Olsztyn, Poland; 5 Institute of Natural Fibres and Medicinal Plants – National Research Institute, Wojska Polskiego 71B, 60-630 Poznań, Poland; 6 Department of Animal Breeding, Institute of Animal Sciences, Warsaw University of Life Sciences, Ciszewskiego 8, 02-786 Warsaw, Poland

**Keywords:** Antimicrobials, Bacteria, Pathogens, Microbiology, Molecular biology, Physiology, Biomarkers

## Abstract

No study has demonstrated the use of dietary *Cannabis*-derived cannabidiol (CBD) to alter the stress response in chickens or examined its effects on meat volatile compounds (VOCs). Here, we subjected chickens to dysbiosis via *C. perfringens* infection or *Escherichia coli* lipopolysaccharide (LPS) treatment and investigated the potential link between meat VOCs and cecal bacterial activity and the ameliorative effect of CBD. The cecal bacterial production of short-chain fatty acids (SCFAs) was closely correlated with meat VOCs. CBD supplementation reduced the formation of breast meat spoilage VOCs, including alcohols, trimethylamine and pentanoic acid, in the challenged birds, partly by decreasing cecal putrefactive SCFA production. Meat VOC/cecal SCFA relationships differed according to the challenge, and CBD attenuated the effects of *C. perfringens* infection better than the effects of LPS challenge on meat VOCs. These findings provide new insights into the interactions among bioactive agent supplementation, gut microbiota activity and meat properties in birds.

## Introduction

Broiler chickens are intensely selected, mostly for increased weight gain and feed efficiency. This selection has resulted in modern lines of poultry with significantly improved growth rates but whose health status is compromised in most key areas, including inflammation, gut barrier function failure and dysbiosis. Particularly unfavorable for the host is dysbiosis, which is an imbalance in the gut microbiota favoring pathogenicity to the host that impairs gastrointestinal tract function, including intestinal barrier function; increases the risk of bacterial translocation; and can lead to muscle abnormalities^1^. It has been reported that pathogenic stimuli disrupt the homeostasis among metabolism (digestion and absorption), the immune system, gut function and the composition and activity of microbiota in the gut^2^. This disruption, in turn, may lead to the deterioration of meat quality as well as the development of a number of muscle abnormalities as a result of metabolic disturbance^3^. As gut barrier function is a primary component of the innate immune response, deeper investigation into the possibility of modulating the initial response to challenge, which in turn may enable the improvement of its further contributions to overall meat quality, is needed^4^. Although clear links among pathogen invasion, gut barrier function, the gut microbiota and meat properties in birds have not yet been elucidated, a growing body of evidence indicates that such an axis exists, which warrants further investigation^5,6^. In the optimal condition of rearing birds (with no induced inflammation), Wang et al.^7^ used a chicken model to demonstrate that supplementation of diets with different strains of probiotic bacteria can affect the growth of microbial populations and the concentration of short-chain fatty acids (SCFAs) in the cecum, which was strongly positively correlated with digestive enzyme activities and significantly affected many volatile compounds, which consequently affected meat flavor components in the breast meat of birds. In that study, dietary intervention exerted significant changes in the concentrations of acetate and butyrate, which are the major SCFAs produced in the caeca. In another study^8^, it was found that prebiotic (galacto-oligosaccharides or xylo-oligosaccharides) supplementation of the chicken diet caused changes in the global metabolome of the cecum through microbiota metabolites, including SCFAs, which significantly affected the flavor of chicken meat. These changes were likely due to induced changes in the gene expression associated with the pathways of fatty acid accumulation, including lipolysis in adipocytes and the adipocytokine signaling pathway. Sun et al.^9^ found that flavor compounds, including VOCs, of chicken meat can be even significantly affected depending on diet or living conditions due to the differences in the formation of flavor precursors or due to the metabolism and degradation of aromatic compounds caused by cecal microbiota. Eventually, supplementation with chicken diet hemp seeds not only caused a reduction in coliform count and promoted the proliferation of *lactobacilli* in the cecum but also significantly reduced serum lipids, including triglyceride, LDL and total cholesterol concentrations, as well as serum enzymes, such as AST and ALT, which are all associated with meat quality^10^. Taken together, these results indicate that the fermentation process in the chicken gut (mainly in the ceca as a main site of fermentation in the bird’s gut) can affect meat indices of quality, including VOCs, mainly as a result of changes in bacterial composition and metabolites. However, an even stronger relationship between host-gut functional status and meat quality can be expected under pathogen stimuli since it leads to fermentation disturbance, as evidenced in different models^11,12^. Lucke et al.^13^ showed that challenging chicken *E. coli*-LPS provoked changes in the bacterial composition of the cecum and increased the concentrations of acetate, butyrate and total SCFAs, suggesting that it could also have an impact on meat properties. In line with this, it was shown that LPS challenge had a significantly negative effect on chicken meat quality^14^. In a study on a meat duck model, it was found that under *E. coli*-LPS inflammation, there were significant alterations in the cecal microbiota as well as produced SCFAs^15^. In a study on a chicken model, infection with *C. perfringens* caused an increase in the host gut abundance of several foodborne pathogens, including *C. jejuni*, *E. coli* and *L. monocylogenes,* and affected the transcript levels of a number of genes regulating host metabolism^16^. In another study, a close correlation between *cecal C. perfringens* and *C. jejuni* was shown, indicating a potential influence on the final product quality^17^. Eventually, in our recent study on a turkey model, we showed that challenging bird *C. perfringens* had a significant effect on the sarcoplasmic protein profile of breast muscle, indicating strong linkage between *C. perfringens* infection and the involvement of glycolytic enzymes in cell metabolism in turkeys^18^.

Recently, considerable attention has been given to investigating the use of bioactive substances in poultry nutrition to improve avian welfare and ensure final product safety and quality^19^. The preventive use of antibiotics and antibiotic-based growth promoters as feed additives was restricted in the European Union (EU) in 2006 (Regulation [EC] No. 1831/2003). Therefore, alternative approaches that might effectively downregulate inflammatory processes in broilers raised under commercial conditions have been investigated. The use of bioactive compounds (natural additives) in poultry feed is among the investigated potential alternatives to the antibiotic-based approach. Such additives might improve certain aspects of avian wellbeing and provide final products that are safe for consumers^20,21^. One phytogenic substance that exhibits the potential to beneficially modulate gut health and functionality is cannabidiol (CBD) obtained from hemp, which has recently attracted considerable attention. According to EU regulations^22^, *Cannabis sativa* plants can be legally registered for cultivation when their tetrahydrocannabinol content is less than 0.2% (w/w).

Reports on the therapeutic potential of CBD have led to many important discoveries in medicine. The biological activity of CBD is mediated by the activation of cannabinoid receptor types 1 and 2 of the endocannabinoid system, which are centrally and peripherally located throughout the body. Therefore, CBD may exhibit inflammation-regulating biological activity by binding to these receptors with high affinity. Because both of these receptors function in processes related to the maintenance of homeostasis, including food intake regulation, nausea/emesis, gastric secretion, gastroprotection, gut motility, ion transport, visceral sensation, intestinal inflammation and cell proliferation in the gut^23^, regulating them may be an effective intervention method to manage the initial stage of inflammation in the host, which could in turn affect metabolic processes contributing to meat features associated with quality, as has been shown in various models^24–26^. Since the metabolism of key elements, including peptides, fatty acids, and amino acids, in the host depends on gut health conditions, understanding the relationships of these elements with the taste and flavor of meat will provide new opportunities to meet consumer demands^27^. Therefore, in the present study, we investigated the effects of CBD on meat volatile organic compounds (VOCs) and gut microbiota activity in chickens reared under optimal conditions or subjected to *C. perfringens*- or *E. coli*-induced inflammation. The specific aims were (i) to characterize the potential correlation between meat characteristics, particularly VOCs, and gut microbiota activity assessed based on the main microbial metabolites (SCFAs); (ii) to investigate the influence of *C. perfringens* and *E. coli* lipopolysaccharide (LPS) challenge on microbiota activity in the chicken ceca; and (iii) to determine the potential of CBD to modulate gut bacterial activity in birds exposed to different conditions and examine its association with meat characteristics.

We hypothesized that pathogen-induced changes in gut microbiota activity would affect meat VOCs, whereas CBD, because of strong microbiota–gut–host interactions, would ameliorate the negative impacts of pathogen challenge on meat properties.

## Results and discussion

### Basic chemical composition of breast muscle

The data regarding the chemical composition of the breast muscle obtained in the present study (Table 1) indicated that the applied treatments did not significantly affect the ash and protein levels in the breast meat (P > 0.05). However, it did affect the fat content in the breast muscle, which was significantly higher in birds supplemented with CBD and challenged with either *C. perfringens* or LPS than in the control and CBD-supplemented birds (P < 0.05). The dry matter content in the breast muscle was significantly higher in the CBD + *C. perfringens* group than in the CON group (P < 0.05). These findings confirm that mild infection with *C. perfringens* can occur without causing visible abnormalities in the muscle tissue, indicating that such meat poses a potential threat as a vector for the delivery of pathogens to the food chain^28^. In the present study, CBD residue in chicken meat was not analyzed; however, based on our previous study^29^, feeding diets over 35-day period contained the same CBD extract as in the present study, but at the inclusion level of 15 g/kg diet, the CBD concentration in the breast muscle was 141.54 ± 95.54 ng/g (on a dry matter basis).

**Table 1:** Chemical composition of chicken breast muscle (%) in response to treatments.

Index	Treatment group^1^						SEM	P value
	CON	CBD	*C. perfringens*	LPS	CBD + *C. perfringens*	CBD + LPS		
Total protein	23.3	23.9	22.9	22.6	22.1	21.6	0.237	ns
Fat	1.43^a^	1.51^a^	1.87^ab^	3.02^abc^	3.70^c^	3.42^bc^	0.258	*
Ash	1.71	1.64	0.75	1.82	1.72	1.9	0.037	ns
Dry matter	25.2^a^	25.6^ab^	25.4^ab^	26.1	27.1^b^	26.3^ab^	0.198	*

^1^CON: received the basal diet over the entire period of the experiment and no challenge; CBD: received the CON diet supplemented (on top) with 30 g/kg *Cannabis sativa* extract; *C. perfringens* and LPS: received the CON diet and subjected to *C. perfringens* and *E. coli* LPS challenge, respectively; CBD + *C. perfringens* and CBD + LPS: received the CBD diet and subjected to *C. perfringens* and *E. coli* LPS challenge, respectively. ^a–c^Means within the same line followed by different superscript letters are significantly different (P < 0.05), and ns indicates no significant difference. The data are the mean of 8 replicates, and the variability is described as the standard error of the mean (SEM).

### Effect of treatment on collagen content in breast meat

**Figure 1:**
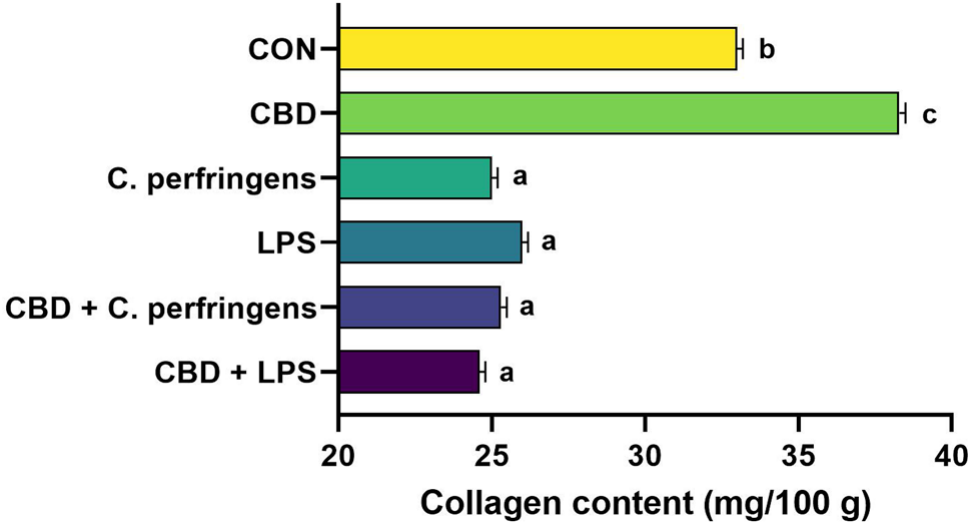
Collagen content in chicken breast muscle. CON: received the basal diet over the entire period of the experiment and no challenge; CBD: received the CON diet supplemented (on top) with 30 g/kg *Cannabis sativa* extract; *C. perfringens* and LPS: received the CON diet and subjected to *C. perfringens* and *E. coli* LPS challenge, respectively; CBD + *C. perfringens* and CBD + LPS: received the CBD diet and subjected to *C. perfringens* and *E. coli* LPS challenge, respectively. ^a–c^Different letters indicate significant differences (P < 0.001). The error bars indicate the standard error values for the 8 chickens in each treatment group.

Compared with the other birds, the birds challenged with either *C. perfringens* or LPS exhibited significantly lower collagen contents in their breast muscle tissue (P < 0.001). The collagen content was the highest in the CBD group, followed by the CON group (Fig. 1). The collagen reductions in all groups of challenged chickens could have resulted from a compromised supply caused by the failure of gut function due to increased activity of collagen-degrading enzymes in the gut tissue. A similar response was found in our previous study, in which collagen disruption occurred in chicken gut tissue due to challenge^29^. In contrast, an increased collagen content in breast muscle has been linked with fibrosis in chickens^30^. However, because the increased collagen content in the present study was found in both the CON- and CBD-supplemented groups, we excluded the possibility that CBD supplementation was associated with muscle abnormalities in our study.

### Differences in breast meat VOCs between groups

The results of VOC profiling are presented in Table 2. Fifteen characteristic compounds were identified. Four compounds were recognized as alcohols, five as aldehydes, two as furans, one as a ketone, one as an acid, one as an amine and one as a terpene. Two alcohols (ethanol and 1-propanol), three aldehydes (propanal, 2-methylpropanal, and 2-methylpentanal), one amine (trimethylamine), one acid (pentanoic acid) and one terpene (terpinolene) were found in the CON group. Trimethylamine, ethanol and 2-methylpropanal levels were lowest in the CON group (P < 0.05), whereas the terpinolene content was the highest in the CON group (P < 0.05). The VOCs detected only in the CBD + *C. perfringens* and CBD + LPS groups were 2-propanol and 2-butylfuran, whereas 2-propionylpyrrole was detected only in the CBD + LPS group. In contrast, both propanal and pentanoic acid were absent only from the challenged groups supplemented with CBD (CBD + *C. perfringens* and CBD + LPS). 2-Methylpropanal was not present in the meat of chickens that received CBD but was present in the meat of the remaining chickens. Overall, the compounds identified in breast meat have been reported to be common in chicken meat^31^. These findings together with those regarding the basic chemical composition of breast muscle indicated that treatment in the present study had a rather moderate effect on the VOC composition of the meat. The main changes in the experimental group were associated with higher concentrations of trimethylamine, ethanol and 2-methylpentanal, which were significantly elevated in all treatments when compared meat from the CON group. The concentrations of these compounds in meat increase with storage time as a result of proteolytic activity, and they are indicative of meat spoilage^32^. These results may suggest that the applied treatments contributed to changes in the meat VOC composition. Certain VOCs could act as indicators of potential meat spoilage. In the present study, we found that 2-propanol was present only in meat from chickens both fed CBD and challenged (*C. perfringens* and LPS). The detection of this compound only in these two groups indicates a potential interaction between CBD and the applied challenges in modulating meat VOCs. Alcohols, including 2-propanol, have been considered spoilage markers in chicken meat and are formed by the reduction of aldehydes derived from lipid oxidation^33^. This agrees with our results, as we found that the fat content in the breast meat was significantly elevated in the CBD + challenge groups.

**Table 2:** Concentrations of volatile compounds in chicken breast meat (relative peak area with the MXT-5 column).

Characteristic			Treatment group^2^						
Possible matching compound^1^	Chemical group of compound	Sensory descriptor	CON	CBD	*C. perfringens*	LPS	CBD + *C. perfringens*	CBD + LPS	P value
Trimethylamine	Amine	Ammoniacal	2.49 ± 0.48^a^	4.27 ± 0.97^bc^	4.32 ± 0.7^bc^	3.14 ± 0.24^ab^	4.90 ± 0.38^c^	3.78 ± 1.85^abc^	< 0.05
Ethanol	Alcohol	Alcoholic	22.70 ± 8.34^a^	46.11 ± 2.06^b^	41.99 ± 0.7^b^	43.28 ± 0.77^b^	41.54 ± 0.20^b^	55.76 ± 5.79^c^	< 0.05
2-Propanol	Alcohol	Alcoholic	nd	nd	nd	nd	9.06 ± 0.90	7.86 ± 0.36	ns
Propanal	Aldehyde	Ethereal	8.49 ± 2.61^a^	11.85 ± 0.18^b^	12.79 ± 0.55^b^	7.42 ± 0.55^a^	nd	nd	< 0.05
2-Methylpropanal	Aldehyde	Burnt	1.57 ± 0.74	nd	2.14 ± 0.67	1.65 ± 0.58	nd	nd	ns
1-Propanol	Alcohol	Alcoholic	9.90 ± 0.53^d^	7.30 ± 1.81^c^	4.92 ± 0.16^b^	4.36 ± 0.30^ab^	5.86 ± 0.25^bc^	3.27 ± 1.14^a^	< 0.05
2-Methylfuran	Furan	Alliaceous	nd	nd	3.05 ± 0.12	nd	3.06 ± 2.25	2.04 ± 0.15	ns
But-(E)-2-enal	Aldehyde	Floral	nd	2.03 ± 0.58	nd	3.59 ± 1.34	nd	nd	ns
(E)-2-penten-1-ol	Alcohol	Grassy	nd	nd	2.45 ± 0.46	nd	nd	nd	ns
2-Methylpentanal	Aldehyde	Earthy	2.42 ± 0.04^a^	4.38 ± 0.14^b^	7.96 ± 0.07^e^	5.23 ± 0.28^c^	7.41 ± 0.30^d^	8.02 ± 0.35^e^	< 0.05
2-Butylfuran	Furan	Spicy	nd	nd	nd	nd	3.83 ± 0.15	4.11 ± 0.14	ns
Pentanoic acid	Acid	Beefy	2.40 ± 0.49^a^	2.69 ± 0.54^a^	4.31 ± 0.30^b^	2.87 ± 0.04^a^	nd	nd	< 0.05
2-Priopionylpyrrole	Ketone	Roasted	nd	nd	nd	nd	nd	1.52 ± 0.19	ns
Benzeneacetaldehyde	Aldehyde	Floral	nd	nd	1.59 ± 0.03^b^	1.03 ± 0.10^a^	nd	nd	< 0.05
Terpinolene	Terpen	Fruity	3.13 ± 0.77^b^	2.87 ± 0.10^b^	2.85 ± 0.17^b^	1.34 ± 0.09^a^	1.52 ± 0.19^a^	1.28 ± 0.04^a^	< 0.05

^1^To elute from the MXT-5 column. ^2^CON: received the basal diet over the entire period of the experiment and no challenge; CBD: received the CON diet supplemented (on top) with 30 g/kg *Cannabis sativa* extract; *C. perfringens* and LPS: received the CON diet and subjected to *C. perfringens* and *E. coli* LPS challenge, respectively; CBD + *C. perfringens* and CBD + LPS: received the CBD diet and subjected to *C. perfringens* and *E. coli* LPS challenge, respectively. ^a–e^Means within the same line followed by different superscript letters are significantly different (P < 0.05), and ns indicates no significant difference. The data are the mean ± standard error (SE) of 8 replicates. nd: not detected.

**Figure 2:**
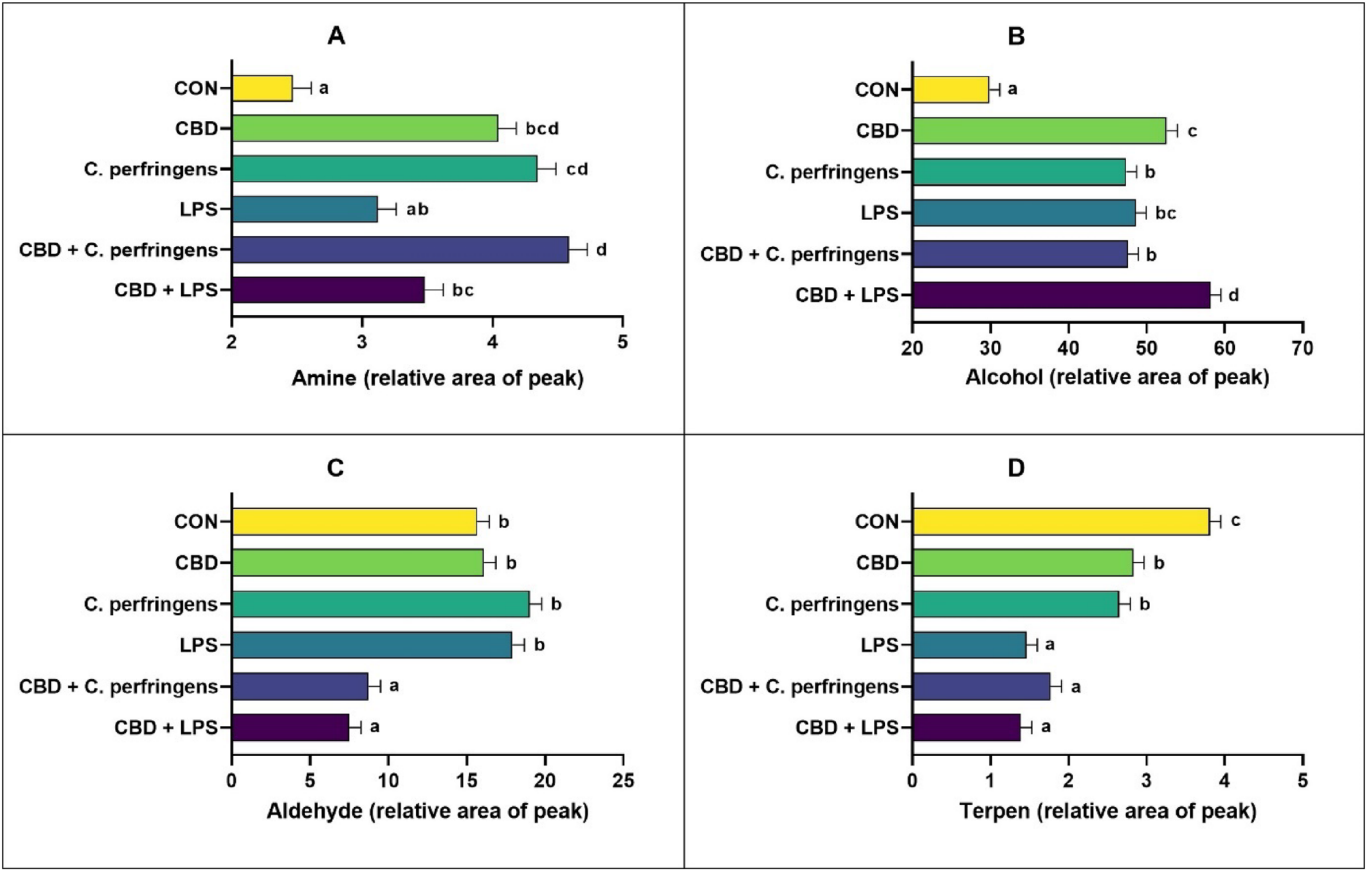
Main groups of chemical compounds detected in chicken breast meat. CON: received the basal diet over the entire period of the experiment and no challenge; CBD: received the CON diet supplemented (on top) with 30 g/kg *Cannabis sativa* extract; *C. perfringens* and LPS: received the CON diet and subjected to *C. perfringens* and *E. coli* LPS challenge, respectively; CBD + *C. perfringens* and CBD + LPS: received the CBD diet and subjected to *C. perfringens* and *E. coli* LPS challenge, respectively. (a–d) Different letters indicate significant differences (P < 0.001). The error bars indicate the standard error values for the 8 chickens in each treatment group.

Regarding the chemical groups of compounds (Fig. 2), the levels of amine compounds in breast meat (Fig. 2a) were significantly elevated in all CBD-supplemented groups and did not differ between the CON and LPS groups (P < 0.001). The alcohol content in meat (Fig. 2b) was significantly higher in all treatment groups than in the CON group (P < 0.001). In contrast, the levels of aldehyde in the meat were significantly lower in the CBD + LPS and CBD + *C. perfringens* groups than in the remaining groups (Fig. 2c; P < 0.001). Terpene compounds were detected at the highest level in the CON group, while the levels were significantly lower in the CBD and LPS groups and were lowest in the LPS, CBD + *C. perfringens* and CBD + LPS groups (P < 0.001). Among other components, aldehydes are particularly important compounds that contribute to meat spoilage. The microorganism family responsible for the production of the most aldehydes is *Enterobacteriaceae*^32^, to which a number of common poultry pathogens belong. Because a natural consequence of pathogen challenge (with *C. perfringens* or *E. coli* LPS) is dysbiosis in the bird gut, we speculate that in the present study, the increased concentration of aldehydes in chicken meat was due to negative shifts in the pathogenic bacteria responsible for producing such components, as reported in a previous study^32^ investigating the contributions of spoilage-related microorganisms to chicken meat properties during storage. Nevertheless, treatment with CBD counteracted the effect of challenge on aldehyde production, suggesting that CBD affects the gut microbiota and meat properties. A similar result was clearly obtained in a study by Pasquali et al.^24^, in which the total numbers of *Enterobacteriaceae* and coliforms were significantly reduced by 2.3 log CFU/g and 1.6 CFU/g meat sample as a result of supplementation with a *Cannabis*-derived extract containing 0.3 mg/ml CBD.

### PCA

**Figure 3:**
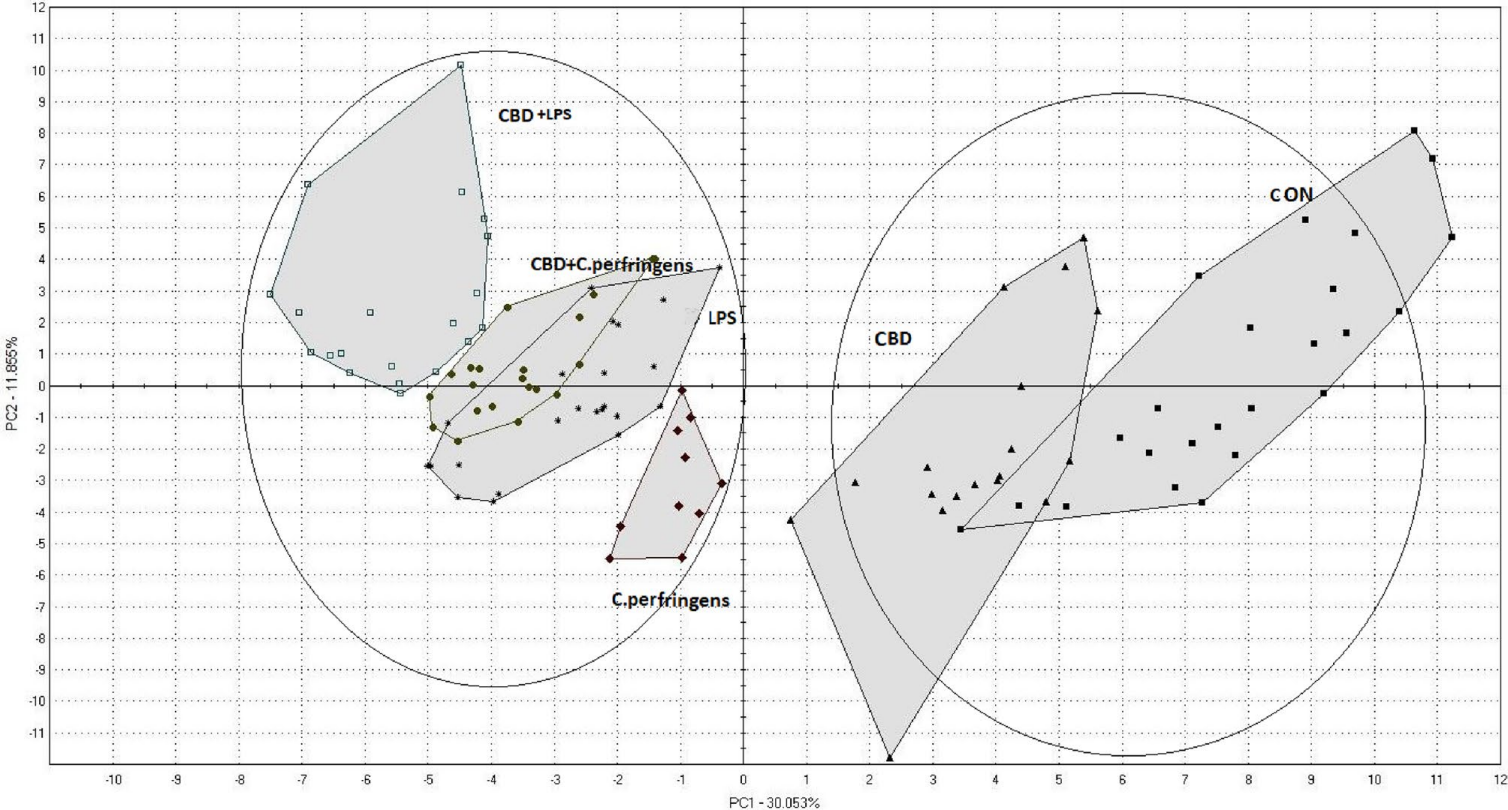
PCA of volatile compounds in chicken breast meat. CON: received the basal diet over the entire period of the experiment and no challenge; CBD: received the CON diet supplemented (on top) with 30 g/kg *Cannabis sativa* extract; *C. perfringens* and LPS: received the CON diet and subjected to *C. perfringens* and *E. coli* LPS challenge, respectively; CBD + *C. perfringens* and CBD + LPS: received the CBD diet and subjected to *C. perfringens* and *E. coli* LPS challenge, respectively.

The variance analysis results are presented in a score plot (Fig. 3). PCA indicated the differences in the volatile components among the six analyzed groups. The principal component on the vertical axis explained 30% of the data variance, while that on the horizontal axis explained 11.86%. The VOC profile of the CON group was in the same area as that of the CBD group, which suggested similar aromas in these groups. However, the *C. perfringens*, LPS, CBD + *C. perfringens* and CBD + LPS groups plotted in separate areas, which indicated that their compositions were different from those of the other groups.

The aroma of chicken meat plays a key role in its quality, determining its overall acceptance by consumers. Therefore, any intervention aimed at affecting meat quality must ensure that the final product maintains an acceptable quality. In the present study, due to the potential threat of the applied challenges (*C. perfringens* and LPS), we could not evaluate the sensory characteristics of the meat based on the opinions of panelists. Instead, we analyzed the VOC profiles of the breast meat. Our data indicated that treatment with CBD did not compromise the meat quality determined based on the VOC profile since the CBD group clustered with the CON group in PCA. Similarly, Skřivan et al.^34^ did not find significant differences in chicken meat quality between a nonsupplemented group and a group supplemented with 150 g/kg feed of expelled hemp seed containing 170 mg/kg CBD. Interestingly, the only group with a different VOC profile was the *C. perfringens* group, indicating a strong impact of *C. perfringens* infection on meat properties. This could be attributed to the fact that *C. perfringens* can affect lipid metabolism via downregulation of the expression of fatty acid catabolism-related genes, including peroxisome proliferator activated receptor-alpha, carnitine palmitoyl transferase 1 and acyl CoA oxidase 1^35^, which play important roles in lipid metabolism and thus strongly contribute to meat sensory properties. Our data also indicated that the meat VOC response differed significantly depending on the challenge factor.

### Correlations between VOCs in breast meat and cecal bacterial activity

Unlike others who have investigated changes in bacterial composition^32^, herein, we investigated changes in the main microbial metabolites as important indicators that have an effect on the health quality of animal products. In the current study, we investigated the correlations between VOCs and bacterial activity based on the concentrations of SCFAs in the ceca of birds. SCFAs are the main end-products of bacterial fermentation, and because of their high density in the gut, they are important environmental determinants of host–microbiota interactions^36^. Additionally, acids, which are the predominant group of VOCs, strongly contribute to flavor development due to their intense odors and relatively low threshold values^7^. Any factors that can disturb host–microbiota equilibrium, i.e., pathogen stimuli, can affect meat properties. The associations between VOCs in breast meat and SCFA concentrations in chicken ceca are shown in Supplementary Table S1a and b. In the CON group, there were strong positive correlations between C3 and propanal and between C3 and 2-methylpropanal (r = 0.794 and r = 0.754, respectively) and a strong negative correlation between C3 and 1-propanol (r = − 0.896). A positive correlation between C2 and trimethylamine (r = 0.728) and a negative correlation between C2 and ethanol (r = − 0.779) were found in the CBD group (Supplementary Table S1a). There was also a negative correlation between C2 and pentanoic acid (r = − 0.815) in the LPS group and a negative correlation between C2 and 2-methylpentanal (r = − 0.751) in the CBD + *C. perfringens* group (Supplementary Table S1b). Butyric acid was negatively correlated with 1-propanol (r = − 0.744) in the CON group, positively correlated with trimethylamine (r = 0.800) in the CBD group (Supplementary Table S1a) and negatively correlated with ethanol (r = − 0.719) in the CBD + *C. perfringens* group (Supplementary Table S1b). The SCFAs produced by cecal bacteria, including C2, C3 and C4, are major components of the SCFA profile in poultry species; therefore, they may contribute to meat features to the greatest extent among the components. The present results showed strong correlations between major SCFAs and alcohol-, trimethylamine- and pentanoic acid-group VOCs in breast meat. Trimethylamine is formed from protein and amine degradation and is considered a biomarker of the loss of muscle-based food freshness and safety^37^, whereas pentanoic acid and other metabolites have been reported to be the best indicators of flavor deterioration in meat products^38^. In line with this evidence, the most obvious association between meat VOCs and SCFAs was the finding in relation to C2 changes (correlated with changes in meat spoilage markers), which indicated the particular contribution of C2 to meat properties. Dietary supplementation with CBD in *C. perfringens*-challenged chickens caused a negative correlation between C4 and the formation of alcohol-derived markers of meat spoilage. Another potential mechanism mediating the meat VOC response to changes in SCFA production is associated with branched SCFAs (C4i and C5i), which are formed from branched-chain amino acids by bacterial fermentative activity using protein-derived substrates^39^. In our study, we found a negative correlation between C4i and propanal (r = − 0.727) in the CBD group (Supplementary Table S1a), a positive correlation between C4i and trimethylamine (r = 0.781) in the LPS group and positive correlations between C4i and trimethylamine and between C4i and 2-propanol (r = 0.840 and r = 0.808) in the CBD + LPS group (Supplementary Table S1b). There were no correlations between C5i and VOCs in the CON group. These findings indicate that CBD affects the formation of meat VOCs through the modulation of branched SCFAs. This is because under the optimal conditions, there was no correlation between C4i and C5i in the CON group, while a significant correlation was found when *C. perfringens* or LPS challenge was applied. Moreover, we found a negative correlation between C5i and terpinolene (r = − 0.756) in the CBD group. There was also a negative correlation between C5i and trimethylamine (r = − 0.750; Supplementary Table S1a) in the *C. perfringens* group, and there was a strong positive correlation between C5i and trimethylamine and between C5i and 2-propanol (r = 0.840 and r = 0.808) in the CBD + LPS group (Supplementary Table S1). These data seem to confirm (as discussed above) that VOC formation differs depending on the challenge factor applied since C5i and trimethylamine were negatively correlated in the *C. perfringens* group but positively correlated in the CBD + LPS group. Terpenes, including terpinolene, are produced by cannabis plants and are responsible for the plants’ characteristic flavors; thus, they may affect meat VOCs during CBD treatment^40,41^. Furthermore, our study revealed that C5 was positively correlated with propanal, 2-methylpropanal and terpinolene (r = 0.777, r = 0.912 and r = 0.961) in the CON group; positively correlated with 2-methylfuran (r = 0.743) in the *C. perfringens* group (Supplementary Table S1a); and positively correlated with trimethylamine, 2-methylpropanal, and but-(E)-2-enal (r = 0.839, r = 0.861 and r = 0.840, respectively; Supplementary Table S1b). This response clearly indicates that differences in VOCs related to SCFAs exist under different conditions. Valeric acid is produced by specific members of the cecal microbiota and exerts beneficial effects on the host gut^42^. In line with this characteristic, in the CON group, valeric acid had strong positive correlations with meat VOCs, including terpinolene, which are known to have antibacterial activity against poultry pathogens^43^; in contrast, *C. perfringens* challenge resulted in the accumulation of meat spoilage markers, including trimethylamine. In contrast, the effects of putrefactive fatty acids on meat VOCs were mostly associated with LPS challenge (irrespective of CBD supplementation) since we found that PSCFAs were positively correlated with 2-methylpropanal (r = 0.776) and terpinolene (r = 0.728) in the CON group (Supplementary Table S1a), positively correlated with trimethylamine (r = 0.925) in the LPS group, and positively correlated with trimethylamine (r = 0.831) and 2-propanol (r = 0.851) in the CBD + LPS group (Supplementary Table S1b). Finally, the sum of SCFAs, which is indicative of bacterial activity, was negatively correlated with 1-propanol (r = − 0.788) in the CON group, positively correlated with trimethylamine (r = 0.814) and negatively correlated with ethanol (r = − 0.726) in the CBD group (Supplementary Table S1a), and negatively correlated with pentanoic acid (r = − 0.789) in the LPS group and with 2-methylpentanal (r = − 0.806) in the CBD + *C. perfringens* group (Supplementary Table S1b). Overall, these results indicate that under optimal conditions, increased bacterial activity provokes meat spoilage. CBD supplementation counteracts this process and is more effective in downregulating meat spoilage marker formation in birds challenged with *C. perfringens* than in birds challenged with *E. coli* LPS.

## Methods

### Ethical statement

All procedures in the present study were evaluated and approved by the Local Animal Care and Ethics Committee in Olsztyn (UWM), Poland (Resolution No. 3/2021) and were performed in accordance with the principles of the EU (recommendation 2007/526/CE) and the Polish Law on Animal Protection. All procedures in this study complied with the ARRIVE guidelines.

### Materials

#### Chemical composition of *Cannabis* extract

Pursuant to the Act on Counteracting Drug Addiction of 29 July 2005 (Journal of Laws of 2017, item 783), the Institute applied for a permit to cultivate fiber hemp (*C. sativa*). Mayor of the Commune Stęszew (Poland) issued approved decision no. Resolution No. 6180.2.2017 of 10 May 2017. Plants for the experiment were cultivated from certified seeds, and all procedures, including either plant cultivation or the collection of plant material, complied with institutional, national, and international guidelines and legislation. The plats that were used for extract preparation were EU-registered (Research Centre for Cultivation Testing, Polish National List of Variety (NLI), Tygra no. R1865) and had a cannabinoid content below 0.2%. In the present experiment, the desired result of the extraction was to obtain the highest concentration of CBD in the extract, which increased the content of other cannabinoids, including tetrahydrocannabinol in the final extract. Consequently, considering the inclusion level (30 g/kg diet) of CBD extract in the diet, the final concentrations were 0.147 g tetrahydrocannabinol per 1 kg of feed (0.015%) and 3.6 g CBD per 1 kg feed (0.36%).

**Figure 4:**
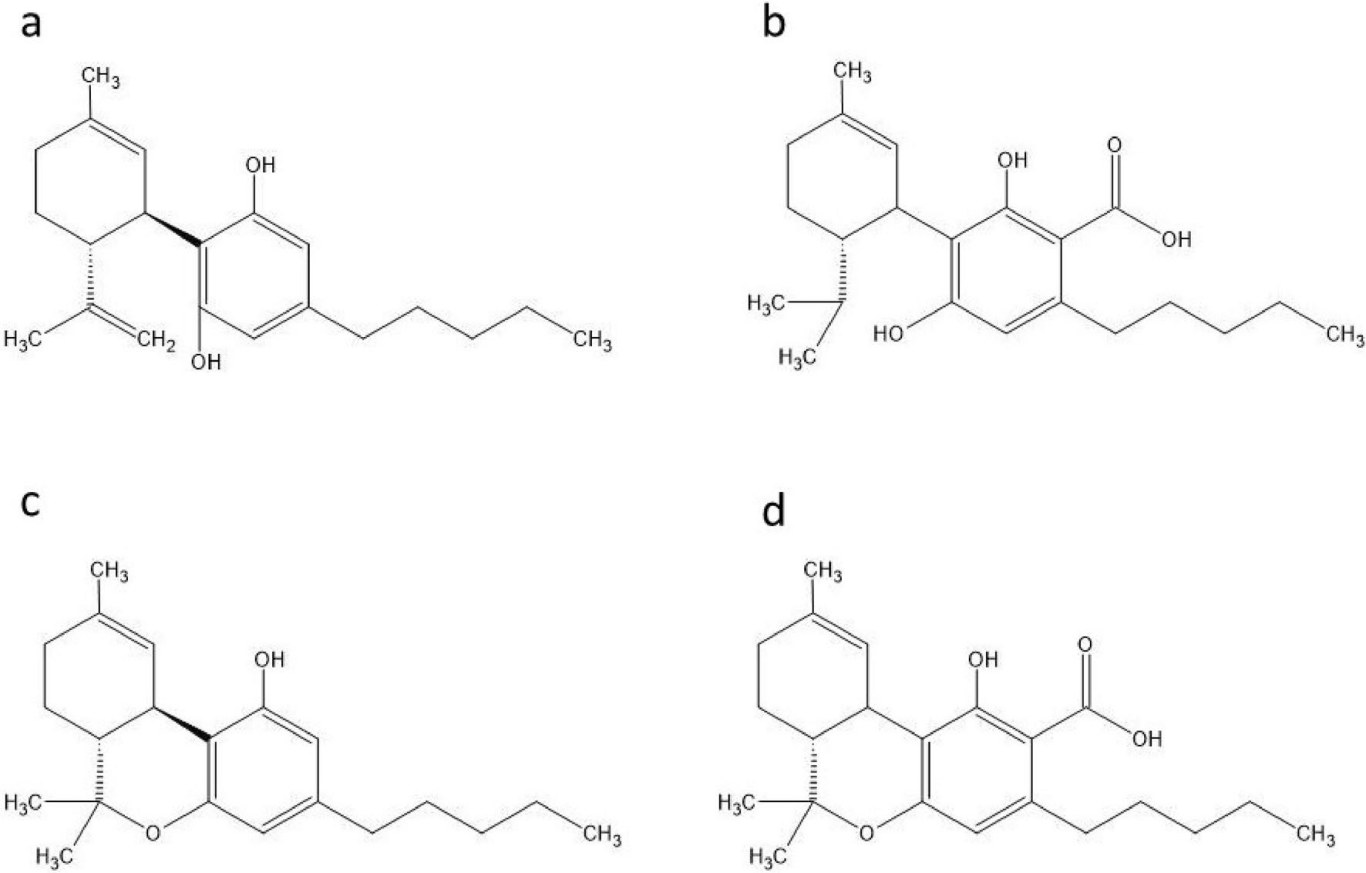
Molecular structures of cannabidiol (**a**), cannabidiol acid (**b**), tetrahydrocannabinol (**c**) and tetrahydrocannabinol acid (**d**). *Source* Thomas and ElSohly^53^.

Hemp panicles obtained from the Institute of Natural Fibers & Medicinal Plants (INF&MP-NRI, Poznań, Poland) were used for supercritical carbon dioxide extraction (pressure: 250 bar, temperature: 60 °C, flow rate: 40 kg CO_2_/1 kg of spent hemp). Voucher specimens were identified by the authors and deposited in the INF&MP-NRI. The crude extract was dissolved in ethanol, and the resulting solution was frozen at − 30 °C for 48 h. Next, the solvent was filtered through paper in a precooled Büchner funnel. The filtrate was collected and subsequently evaporated to remove the alcohol under vacuum at 40 °C. After the evaporation process, the hemp extract contained 12% CBD, 0.49% tetrahydrocannabinol and 0.38% tetrahydrocannabinolic acid, as determined by HPLC. The molecular structures of the bioactive compounds are provided in Fig. 4.

#### Birds, housing, diets, and experimental design

**Figure 5:**
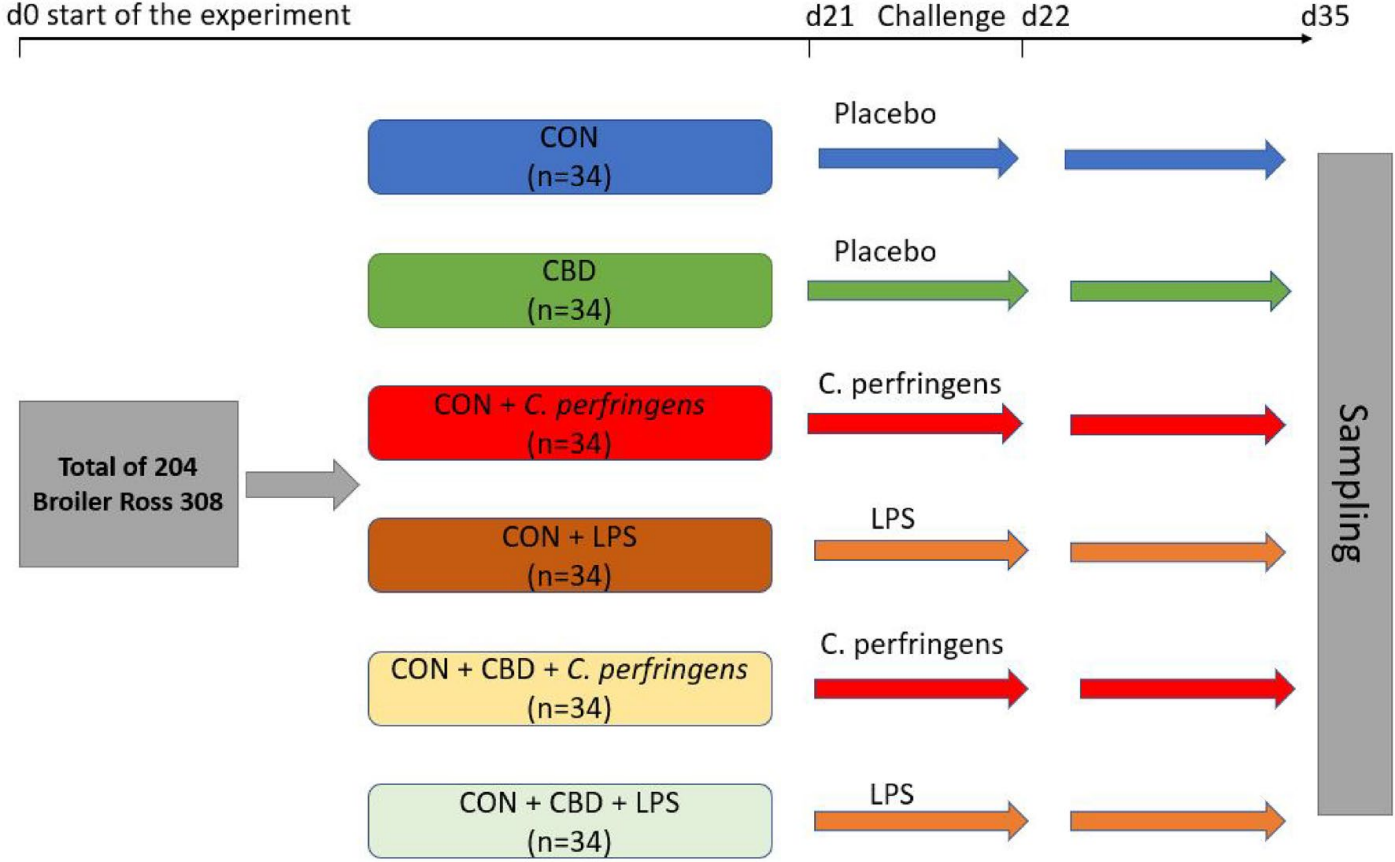
Scheme of the experimental treatments and applied challenge. CON: received the basal diet over the entire period of the experiment (until 35 days of age) and no challenge; CBD: received the CON diet supplemented (on top) with 30 g/kg *Cannabis sativa* extract; *C. perfringens* and LPS: received the CON diet and subjected to *C. perfringens* and *E. coli* LPS challenge at days 21 and 22 of age, respectively; CBD + *C. perfringens* and CBD + LPS: received the CBD diet and subjected to *C. perfringens* and *E. coli* LPS challenge, respectively.

Meat samples were obtained from chickens. Briefly, a total of 204 Ross 308 male broilers were purchased from a local hatchery on the day of hatching. After arrival at the experimental unit, the chicks were distributed according to average body weight into 6 treatment groups, each of which contained 34 chicks. The birds in each treatment group were maintained on litter in pens and were fed similar to the standard commercial starter (days 0–7) and grower (days 8–35) diets (the same basal diet was used for each treatment) formulated to meet or exceed the nutritional requirements of Ross 308 broilers in accordance with their age^44^ (Table 3). Access to feed and drinking water was unrestricted throughout the experimental period. The conditions of the room, including the temperature, humidity and light cycle, were maintained according to standard management practices for commercial chicken houses. Six treatments were applied. The birds in the control (CON) group received a basal diet over the entire period of the experiment. The birds in the CBD treatment group received the CON diet supplemented (on top) with 30 g/kg *C. sativa* extract, and the birds in the CON + *C. perfringens* and CON + LPS groups (positive control groups) were fed the basal diet and subjected to *C. perfringens* and LPS challenge, respectively. The birds in the CBD + *C. perfringens* and CBD + LPS groups received the same diet as the birds in the CBD treatment group in addition to being subjected to the respective challenges described above (Fig. 5). The diets were cold pelleted using a CL-2 CPM (CA, USA) laboratory pellet mill and were provided ad libitum.

**Table 3:** Composition of basal diets (g/kg as-is, unless indicated otherwise) fed to broilers over a 35-day feeding period.

Component	Starter (days 0–7)	Grower (days 8–35)
Maize	200.0	200.0
Soybean meal	306.9	270.0
Wheat	432.0	463.0
Lard	20.1	31.5
Ronozyme^1^	0.2	0.1
Salt	3.3	3.1
Limestone	15.4	14.0
Monocalcium phosphate	10.8	8.6
Choline chloride	1.0	1.0
DL-Methionine	3.0	2.6
L-Lysine	3.7	2.8
L-Threonine	1.1	0.8
Vitamins + trace minerals^2^	2.5	2.5
**Calculated nutrient density**		
ME (kcal/kg)	2900	3000
Crude protein (g/kg)	22.0	20.5
Crude fiber (g/kg)	2.72	2.65
Crude fat (g/kg)	4.0	5.16
Crude ash (g/kg)	2.7	2.53
Lysine (g/kg)	1.36	1.2
Methionine (g/kg)	0.61	0.55
Met. + Cys (g/kg)	1.0	0.92
Threonine (g/kg)	0.88	0.8
Calcium (g/kg)	0.95	0.85
Available phosphorus (g/kg)	0.4	0.35

^1^Ronozyme WX (Novozymes, Copenhagen, Denmark); 360 FXU/kg diet.

^2^Provided per kg feed: IU: vit. A. 10,000, vit. D3 4500; mg: vit. E 80, vit. B1 1.5, vit. B2 5, biotin 0.12, vit. B6 2.5, vit. B12 0.02, vit. K33, nicotinic acid 50, folic acid 1.1, pantothenic acid 14, choline 200, betaine 160, Mn 120, Zn 100, Se 0.35, Cu 20, Fe 40, J 3; g: Ca 0.6.

### Applied experimental challenges

An LPS challenge was conducted. Briefly, birds were individually weighed after 4 h of feed deprivation, and the birds in the CON + LPS and CBD + LPS groups were orally (*per os*) administered LPS (*Escherichia coli* serotype O55:B5; Sigma Chemical, St. Louis, MO, USA) reconstituted in 0.9% sterile saline (0.5 mg/mL) at 21 and 22 days of age in a dose of 1 mL containing 250 μg/kg body weight LPS^45^. At the same time, (days 21 and 22), the birds in the CON + *C. perfringens* and CBD + *C. perfringens* groups were infected (*per os*) with 1 mL of inoculum (brain–heart infusion medium) containing approximately 10^8^ CFU/mL *C. perfringens* type A strain 56 bacteria according to a previously validated protocol^29^. *C. perfringens* bacteria were obtained from infected chickens in Belgium. The strain used was analytically confirmed to be α-toxin- and NetB toxin-positive and β-toxin- and enterotoxin-negative, as declared by the supplier (Ghent University, Merelbeke, Belgium). The birds in the CON and CBD groups were administered (each of them) the same dose of sterile saline and brain–heart infusion medium with coccidial cocktail (placebo groups) (Fig. 5).

### Sampling procedure

At 35 days of age, all birds were weighed individually, and broilers (n = 8 per group) with body weights similar to the group average were selected (Table 4), electrically stunned (150 mA, 350 Hz) using stunning equipment (Water bath GL-70, Święta Katarzyna, Poland), subsequently decapitated and immediately processed for sampling (the slaughtering procedure was previously approved by the Local Animal Care and Ethics Committee). Immediately after slaughter, the birds were plucked and eviscerated, and the whole right breast muscle (*pectoralis major*) was excised without skin, vacuum-packaged, and stored frozen at − 18 °C for further analyses. From the same birds, the whole intestinal tract was removed, and the digesta from both ceca were collected aseptically from each bird and pooled in test tubes (n = 8 per group). Afterward, the samples were immediately frozen at − 32 °C for SCFA analysis.

**Table 4:** Body weight (kg) of birds at 35 days of life.

CON	CBD	*C. perfringens*	LPS	CBD + *C. perfringens*	CBD + LPS
2184.5	2145.2	1846.3	1910	2064.5	1744.5
2162.6	1927.6	2071.1	2036	1814.7	1974.2
2047.5	1673.9	2214.1	1986	1986.5	1994.3
2194.7	1752.1	2042.5	2163	1937.2	2009.3
2199.6	1861.1	2124.5	2010	1890.4	1966.4
2317.2	1914.6	2046.3	1945	1875.3	2051.6
2361.4	2057.1	2164.7	2041	1966.8	2060.0
2390.4	1999.8	2294.7	2299	1940.7	2196.0
**Mean body weight**					
2232.2	1916.4	2100.5	2048.5	1934.5	1999.5
**SD**					
115.0	155.0	134.7	126.1	76.1	126.4

CON: received the basal diet over the entire period of the experiment and no challenge; CBD: received the CON diet supplemented (on top) with 30 g/kg *Cannabis sativa* extract; *C. perfringens* and LPS: received the CON diet and subjected to *C. perfringens* and *E. coli* LPS challenge, respectively; CBD + *C. perfringens* and CBD + LPS: received the CBD diet and subjected to *C. perfringens* and *E. coli* LPS challenge, respectively.

#### Determination of the basic composition of breast meat

The basic composition characteristics, including the dry matter content and the ether extract, protein and ash levels, in the chicken breast meat were analyzed according to the Association of Official Analytical Chemists^46^ methods. Briefly, the dry matter content was determined by drying the meat samples in an oven at 105 °C for 3 h. The fat content was obtained by the Soxhlet method^47^ with ether extraction. The protein content was measured according to ISO standard 1871:2009^48^ (the Kjeldahl method; FOSS Tecator™ 1035 Analyzer), and the ash content was determined by incinerating individual samples (weighing 3–4 g) in a Heraeus furnace for 12 h at 550 °C. Each analysis was performed in triplicate.

#### Determination of the collagen content in breast meat

The muscle collagen concentration was analyzed based on the hydroxyproline concentration according to a protocol previously described by Gawronska-Kozak^49^ with some modifications. Briefly, the muscle samples were thawed at 4 °C overnight, trimmed of fat and epimysium, and weighed. Then, meat samples of approximately 250 mg were homogenized with a glass homogenizer on ice in 2 mL of cold (4 °C) phosphate-buffered saline (Sigma–Aldrich by Merck). Then, the assay was performed with a Hydroxyproline Determination Kit (Sigma–Aldrich by Merck) according to the instructions. Hydroxyproline (Sigma–Aldrich by Merck) was used to prepare a standard curve. The samples were analyzed at 550 nm using a microplate reader (Multiskan Sky Microplate Spectrophotometer, Thermo Fisher Scientific), and the concentration of collagen was expressed as milligrams of collagen per 100 g of muscle.

#### Analysis of the VOC profile in breast meat

The VOCs in chicken meat were analyzed using a Heracles II electronic nose (Alpha M.O.S., Toulouse, France). The method was adopted from the protocols of Wojtasik-Kalinowska et al.^50^ and Górska-Horczyczak et al.^51^. The electronic nose analysis was based on ultrafast gas chromatography with headspace sampling. The equipment consisted of a detector system with two metal columns of different polarities (nonpolar MXT-5 and slightly polar MXT1701, diameter = 180 µm, length = 10 m) and two flame ionization detectors (FIDs). The Kovats indices were determined based on alkane standards (n-butane to n-hexadecane) (Restek) measured under the same conditions as the samples. VOC identification was conducted using AroChemBase (Alpha MOS Co., Toulouse, France), which contains 44 000 compounds and includes a set of sensory descriptors for each included compound. Approximately 3 g of breast meat (without visible abnormalities or fat tissue) was placed into 20-mL headspace vials, which were capped with Teflon-faced silicone caps. Then, the vials containing the samples were incubated at 55 °C for 900 s under agitation (8.33 Hz). The carrier gas (hydrogen) was circulated at a constant flow rate (1 ml min^−1^). The injector temperature was 200 °C, the injection volume was 2500 µL, and the injection speed was 125 mL/s. The analytes were collected in the trap at 15 °C and then divided and simultaneously transferred into the two columns. The carrier gas was applied at a constant pressure of 80 kPa. The split flow rate was 10 mL/min at the column heads. The temperature program of the oven was set as follows: 60 °C for 2 s, followed by a 3 °C/s ramp to 270 °C, a hold for 20 s, and FID1/FID2 at 280 °C. The samples were analyzed in triplicate.

#### Analysis of bacterial activity

Microbial activity in the cecal digesta was measured based on the concentration of SCFAs, including acetic acid (C2), propionic acid (C3), butyric acid (C4), isobutyric acid (C4i), valeric acid (C5) and isovaleric acid (C5i), using gas chromatography (Shimadzu GC-2010, Kyoto, Japan). All sample preparation steps as well as the conditions in the chromatography system followed a protocol previously reported by Konieczka et al.^52^. The pH of the cecal digesta samples was adjusted to 8.2 with 1 mol/L NaOH to convert the SCFAs to sodium salts. Subsequently, the samples were centrifuged at 10,000×*g* for 10 min at 4 °C. Before loading into the chromatography system, all samples were maintained at 4 °C in an apparatus autosampler. Each SCFA concentration was determined based on the concentration of the internal standard (isocaproic acid) added to each sample. All determinations were performed in duplicate.

#### Statistical analysis

One-way analysis of variance (ANOVA) was conducted using Fisher’s least significant difference test. P < 0.05 was considered to indicate a significant difference. The results were analyzed using Statistica 13.1 (StatSoft Inc., Tulsa, USA). The flavor profile was determined by principal component analysis (PCA) with AlphaSoft Version 8.0. The correlations between microbiota activity and breast muscle indices as well as other experimental data were assessed using Pearson’s correlation, with P < 0.05 indicating a significant correlation.

## Conclusion

The present study demonstrated the existence of close correlations between gut bacterial activity and chicken meat VOCs based on the production of SCFAs. Under the optimal conditions (no challenge), increased production of the main SCFAs, including C2 and C3, was associated with increased formation of spoilage markers in the meat. Dietary supplementation with CBD reduced the formation of selected spoilage VOCs, including some alcohols, trimethylamine and pentanoic acid, in challenged birds. CBD supplementation decreased the production of putrefactive fatty acids, which resulted in decreased production of spoilage VOCs in breast meat. Moreover, our findings indicated that the association between VOCs and SCFAs differed depending on the applied challenge (*C. perfringens* vs. LPS), and under these circumstances, CBD more effectively altered the influence of *C. perfringens* than LPS challenge on meat VOCs. The findings of this study provide a basis for understanding the influence of gut bacterial activity on meat VOCs and the particular role of pathogenic stimuli in this regard. The results also provide new insights into the interactions among bioactive agent application, gut microbiota activity and meat properties in birds. However, due to the complexity of pathogen–host interactions, more research is needed to support the production of improved poultry meat.

## Supplementary Information

Supplementary Table S1.

Supplementary Table S1.

## Data Availability

The datasets used and/or analyzed during the current study are available from the corresponding author upon reasonable request. All data generated or analyzed during this study are included in this published article (and its Supplementary Information files).
