# Supplementary material for: Cannabidiol affects breast meat volatile compounds in chickens subjected to different infection models
Source: Sci Rep. 2022 Nov 7;12:18940. doi: 10.1038/s41598-022-23591-1 (PMC9640543; doi:10.1038/s41598-022-23591-1)
Supplement: Supplementary file 1 — Supplementary Table S1. [file 41598_2022_23591_MOESM1_ESM.docx]

**Supplementary Table S1a.** Calculated Pearson’s correlation coefficients between short-chain fatty acids (SCFAs) in the cecal digesta and breast meat volatile compounds in chickens subjected to different challenges.

| Compound | C2 | C3 | C4i | C4 | C5i | C5 | PSCFAs | Total SCFAs |
| --- | --- | --- | --- | --- | --- | --- | --- | --- |
| CON^1^ |  |  |  |  |  |  |  |  |
| Trimethylamine | -0.613 | 0.261 | 0.499 | -0.352 | 0.389 | 0.236 | 0.418 | -0.557 |
| Ethanol | -0.329 | 0.089 | -0.054 | -0.188 | -0.233 | -0.105 | -0.170 | -0.345 |
| 2-Propanol | nd | nd | nd | nd | nd | nd | nd | nd |
| Propanal | 0.283 | 0.794* | 0.537 | 0.635 | 0.487 | 0.777* | 0.604 | 0.606 |
| 2-Methylpropanal | -0.033 | 0.754* | 0.676 | 0.586 | 0.667 | 0.912* | 0.776* | 0.342 |
| 1-Propanol | -0.494 | -0.896* | 0.003 | -0.744* | 0.031 | -0.562 | -0.106 | -0.778* |
| 2-Methylfuran | nd | nd | nd | nd | nd | nd | nd | nd |
| But-(E)-2-enal | nd | nd | nd | nd | nd | nd | nd | nd |
| 2-Methylpentanal | 0.360 | 0.028 | 0.475 | -0.530 | 0.365 | -0.232 | 0.294 | 0.143 |
| 2-Butylfuran | nd | nd | nd | nd | nd | nd | nd | nd |
| Pentanoic acid | 0.350 | 0.487 | 0.572 | 0.303 | 0.470 | 0.447 | 0.532 | 0.503 |
| Benzeneacetaldehyde | nd | nd | nd | nd | nd | nd | nd | nd |
| Terpinolene | -0.459 | 0.633 | 0.576 | 0.586 | 0.614 | 0.961* | 0.728* | -0.027 |
| CBD^2^ |  |  |  |  |  |  |  |  |
| Trimethylamine | 0.728* | 0.796* | -0.028 | 0.800* | 0.796* | 0.708 | 0.246 | 0.814* |
| Ethanol | -0.779* | -0.459 | 0.411 | -0.568 | 0.507 | -0.332 | 0.331 | -0.726* |
| 2-Propanol | nd | nd | nd | nd | nd | nd | nd | nd |
| Propanal | -0.141 | -0.178 | -0.727* | 0.325 | -0.294 | -0.247 | -0.447 | -0.070 |
| 2-Methylpropanal | nd | nd | nd | nd | nd | nd | nd | nd |
| 1-Propanol | 0.333 | 0.592 | 0.427 | 0.068 | 0.679 | 0.628 | 0.681 | 0.360 |
| 2-Methylfuran | nd | nd | nd | nd | nd | nd | nd | nd |
| But-(E)-2-enal | 0.586 | 0.191 | -0.390 | 0.301 | -0.501 | 0.157 | -0.366 | 0.492 |
| 2-Methylpentanal | 0.416 | 0.261 | -0.165 | 0.043 | -0.001 | 0.070 | -0.031 | 0.337 |
| 2-Butylfuran | nd | nd | nd | nd | nd | nd | nd | nd |
| Pentanoic acid | 0.076 | 0.054 | -0.282 | 0.330 | -0.057 | 0.304 | -0.038 | 0.134 |
| Benzeneacetaldehyde | nd | nd | nd | nd | nd | nd | nd | nd |
| Terpinolene | -0.272 | -0.529 | -0.449 | 0.106 | -0.756* | -0.460 | -0.689 | -0.270 |
| *C. perfringens*^3^ |  |  |  |  |  |  |  |  |
| Trimethylamine | -0.080 | -0.356 | -0.517 | 0.022 | -0.750* | 0.127 | -0.544 | -0.164 |
| Ethanol | 0.029 | 0.130 | 0.410 | 0.251 | 0.325 | 0.330 | 0.438 | 0.101 |
| 2-Propanol | nd | nd | nd | nd | nd | nd | nd | nd |
| Propanal | 0.542 | 0.625 | 0.232 | -0.249 | -0.058 | 0.695 | 0.297 | 0.565 |
| 2-Methylpropanal | -0.069 | -0.059 | -0.113 | -0.525 | -0.053 | 0.312 | 0.043 | -0.119 |
| 1-Propanol | -0.560 | -0.594 | -0.251 | 0.170 | 0.045 | -0.677 | -0.304 | -0.583 |
| 2-Methylfuran | 0.381 | 0.458 | -0.247 | -0.696 | -0.467 | 0.743* | -0.076 | 0.330 |
| But-(E)-2-enal | nd | nd | nd | nd | nd | nd | nd | nd |
| 2-Methylpentanal | -0.190 | 0.396 | -0.313 | -0.309 | -0.349 | 0.211 | -0.224 | -0.133 |
| 2-Butylfuran | nd | nd | nd | nd | nd | nd | nd | nd |
| Pentanoic acid | 0.119 | 0.314 | 0.048 | -0.049 | -0.081 | 0.350 | 0.097 | 0.159 |
| Benzeneacetaldehyde | -0.621 | -0.525 | -0.046 | 0.305 | 0.221 | -0.519 | -0.080 | -0.594 |
| Terpinolene | 0.493 | 0.311 | -0.027 | 0.113 | -0.369 | 0.483 | -0.039 | 0.482 |

^1^ CON: received the basal diet over the entire period of the experiment and no challenge. ^2^ CBD: received the CON diet supplemented (on top) with 30 g/kg *Cannabis sativa* extract. ^3^ *C. perfringens*: received the CON diet, and the birds were subjected to *C. perfringens* challenge. C2: acetate; C3: propionate; C4i: isobutyrate; C4: butyrate; C5i: isovalerate; C5: valerate; PSCFAs: putrefactive SCFAs. nd: not detected. *Significant correlation at P < 0.05.
