# Supplementary material for: Cannabidiol affects breast meat volatile compounds in chickens subjected to different infection models
Source: Sci Rep. 2022 Nov 7;12:18940. doi: 10.1038/s41598-022-23591-1 (PMC9640543; doi:10.1038/s41598-022-23591-1)
Supplement: Supplementary file 2 — Supplementary Table S1. [file 41598_2022_23591_MOESM2_ESM.docx]

**Supplementary Table S1b.** Calculated Pearson’s correlation coefficients between short-chain fatty acids (SCFAs) in the cecal digesta and breast meat volatile compounds in chickens subjected to different challenges.

| Compound | C2 | C3 | C4i | C4 | C5i | C5 | PSCFAs | Total SCFAs |
| --- | --- | --- | --- | --- | --- | --- | --- | --- |
| LPS^1^ |  |  |  |  |  |  |  |  |
| Trimethylamine | 0.304 | 0.568 | 0.781* | 0.317 | 0.833* | 0.839* | 0.925* | 0.451 |
| Ethanol | 0.266 | 0.016 | 0.261 | 0.132 | 0.640 | -0.172 | 0.288 | 0.246 |
| 2-Propanol | nd | nd | nd | nd | nd | nd | nd | nd |
| Propanal | 0.342 | -0.228 | 0.044 | 0.309 | 0.254 | -0.467 | -0.064 | 0.277 |
| 2-Methylpropanal | 0.086 | 0.372 | 0.482 | -0.106 | 0.491 | 0.861* | 0.701 | 0.150 |
| 1-Propanol | 0.163 | -0.486 | -0.337 | 0.089 | -0.346 | -0.538 | -0.465 | 0.026 |
| 2-Methylfuran | nd | nd | nd | nd | nd | nd | nd | nd |
| But-(E)-2-enal | -0.202 | 0.402 | 0.527 | -0.148 | 0.470 | 0.840* | 0.695 | -0.068 |
| 2-Methylpentanal | -0.053 | -0.190 | -0.004 | 0.140 | -0.139 | -0.399 | -0.227 | -0.056 |
| 2-Butylfuran | nd | nd | nd | nd | nd | nd | nd | nd |
| Pentanoic acid | -0.815* | -0.152 | -0.263 | -0.677 | -0.339 | 0.011 | -0.218 | -0.789* |
| Benzeneacetaldehyde | 0.406 | 0.566 | 0.425 | 0.649 | 0.044 | 0.217 | 0.220 | 0.552 |
| Terpinolene | -0.090 | -0.192 | -0.041 | 0.126 | -0.201 | -0.435 | -0.279 | -0.090 |
| CBD + *C. perfringens*^2^ |  |  |  |  |  |  |  |  |
| Trimethylamine | -0.162 | -0.072 | 0.460 | -0.193 | 0.358 | 0.234 | 0.402 | -0.174 |
| Ethanol | -0.107 | 0.225 | 0.519 | -0.719* | 0.601 | 0.047 | 0.517 | -0.265 |
| 2-Propanol | 0.080 | -0.044 | -0.359 | 0.192 | -0.256 | -0.017 | -0.262 | 0.103 |
| Propanal | nd | nd | nd | nd | nd | nd | nd | nd |
| 2-Methylpropanal | nd | nd | nd | nd | nd | nd | nd | nd |
| 1-Propanol | -0.460 | -0.667 | -0.389 | 0.375 | -0.360 | -0.440 | -0.430 | -0.355 |
| 2-Methylfuran | 0.157 | 0.314 | -0.058 | 0.035 | -0.047 | 0.105 | -0.020 | 0.179 |
| But-(E)-2-enal | nd | nd | nd | nd | nd | nd | nd | nd |
| 2-Methylpentanal | -0.751* | -0.731* | 0.216 | -0.344 | 0.243 | -0.575 | 0.073 | -0.806* |
| 2-Butylfuran | -0.130 | 0.352 | 0.082 | -0.359 | 0.120 | 0.181 | 0.137 | -0.166 |
| Pentanoic acid | nd | nd | nd | nd | nd | nd | nd | nd |
| Benzeneacetaldehyde | nd | nd | nd | nd | nd | nd | nd | nd |
| Terpinolene | 0.055 | -0.444 | -0.116 | 0.026 | 0.027 | -0.084 | -0.040 | -0.016 |
| CBD + LPS^3^ |  |  |  |  |  |  |  |  |
| Trimethylamine | 0.176 | 0.131 | 0.840* | -0.025 | 0.827* | 0.550 | 0.831* | 0.181 |
| Ethanol | -0.529 | 0.030 | -0.257 | -0.508 | -0.182 | -0.329 | -0.277 | -0.490 |
| 2-Propanol | -0.160 | 0.307 | 0.808* | -0.421 | 0.942* | 0.472 | 0.851* | -0.116 |
| Propanal | nd | nd | nd | nd | nd | nd | nd | nd |
| 2-Methylpropanal | nd | nd | nd | nd | nd | nd | nd | nd |
| 1-Propanol | 0.231 | -0.121 | 0.055 | 0.325 | 0.071 | 0.117 | 0.091 | 0.223 |
| 2-Methylfuran | -0.336 | -0.581 | -0.324 | -0.258 | -0.309 | -0.434 | -0.395 | -0.385 |
| But-(E)-2-enal | nd | nd | nd | nd | nd | nd | nd | nd |
| 2-Methylpentanal | 0.328 | 0.410 | 0.207 | 0.281 | 0.042 | 0.379 | 0.213 | 0.352 |
| 2-Butylfuran | -0.454 | -0.356 | -0.026 | -0.445 | 0.072 | -0.247 | -0.060 | -0.459 |
| Pentanoic acid | nd | nd | nd | nd | nd | nd | nd | nd |
| Benzeneacetaldehyde | nd | nd | nd | nd | nd | nd | nd | nd |
| Terpinolene | -0.518 | -0.253 | -0.207 | -0.468 | 0.065 | -0.341 | -0.147 | -0.501 |

^1^ Received the basal diet and subjected to LPS challenge. ^2^ CBD + *C. perfringens*: received the CON diet supplemented (on top) with 30 g/kg *Cannabis sativa* extract and subjected to *C. perfringens* challenge. ^3^ Received the CON diet supplemented (on top) with 30 g/kg *Cannabis sativa* extract and subjected to LPS challenge. C2: acetate; C3: propionate; C4i: isobutyrate; C4: butyrate; C5i: isovalerate; C5: valerate; PSCFAs: putrefactive SCFAs. nd: not detected. *Significant correlation at P < 0.05.
